# Supplementary material for: Gene Delivery Mediated by Backbone-Degradable RAFT Copolymers
Source: Biomacromolecules. 2026 Feb 12;27(3):1846–56. doi: 10.1021/acs.biomac.5c01662 (PMC12977016; doi:10.1021/acs.biomac.5c01662)
Supplement: Supplementary file 1 [file bm5c01662_si_001.pdf]

Supporting Information

Gene Delivery Mediated by Backbone-Degradable RAFT Copolymers

Prajakatta B. Mulay[1,#,†], D. Christopher Radford[1,#], Brayan Rondon[2], Bruna Favetta[1], Benjamin S. Schuster[3], Jia Niu,[2,*], Adam J. Gormley[1,*]

[1] Department of Biomedical Engineering, Rutgers, The State University of New Jersey, Piscataway, NJ 08854, USA

[2] Department of Chemistry, Boston College, Chestnut Hill, MA 02467, USA

[3] Department of Chemical and Biochemical Engineering, Rutgers, The State University of New Jersey, Piscataway, NJ 08854, USA

Supplemental Materials and Methods

pDNA Complexation Efficiency Assay:
Stock solutions of pDNA were prepared in sterile-filtered DNAase-free PBS (pH 7.4) at a concentration of 10 ng/µL. Stock solutions of the cationic copolymers were also prepared in the same buffer to the desired concentrations and mixed 1:1 with the pDNA to obtain the desired N/P ratios of 5, 10, and 20 at a final concentration of 5 ng/µL pDNA. Polyplexes were allowed to incubate for 45 minutes at room temperature prior to measurement. Free pDNA remaining after complexation was then quantified using the Quant-iT PicoGreen dsDNA Assay Reagent (Thermo Fisher Scientific)**.** The assay was then preformed according the manufacturer specifications. In brief, the Picogreen reagent was first diluted into sterile filtered DNAase-free PBS (pH 7.4). The polyplex solution was then diluted 5x in the Picogreen stock solution to obtain a final pDNA concentration of 1 ng/µL. Concentration of free pDNA was then quantified using a Spectramax M3 platereader (Ex 480 nm/ Em 525 nm) by comparing against a standard curve of known pDNA concentrations. Complexation efficiency was then calculated as (Total pDNA - Free pDNA)/ Total pDNA.

Gel Electrophoresis:
Gel electrophoresis was performed using 1 wt% agarose gels prepared in 1x Tris-Acetate-EDTA (TAE) buffer (pH 7.4). Polyplexes were prepared as described above for the Picogreen assay. Two µL of SDS-free DNA gel loading dye (Thermo Fisher Scientific) was added to 10 µL each polyplex sample prior to loading the sample in the gel and electrophoresing for 35 min at 120 V in TAE running buffer. The gel was then imaged using an Azure Biosystems 600 Imager.

DLS Characterization:
Stock solutions of pDNA were prepared in sterile-filtered DNAase-free PBS (pH 7.4) at a concentration of 80 ng/µL. Stock solutions of the cationic copolymers were also prepared in the same buffer to the desired concentration and mixed 1:1 with the pDNA to obtain the desired N/P ratios of 5, 10, and 20 at a final concentration of 40 ng/µL pDNA. Polyplexes were allowed to incubate for 45 minutes at room temperature prior to measurement. DLS measurements were performed on a DynaPro Plate Reader III (Wyatt Technologies) and analyzed using the accompanying Dynamics 7.10 software package. For each sample, five repeat acquisitions with five second acquisition time were collected at 25°C.

Image Analysis:

a. Transfection Efficiency: Transfection efficiency was quantified using object-based fluorescence intensity analysis rather than bulk well fluorescence. The 'Total Count' output from GFP and Hoechst target analysis was considered from the raw data file for measuring the transfection efficiency. Celigo 'Total Count' is the number of segmented objects (GFP+ cells or nuclei) detected per well under the defined imaging and analysis settings (provided in the raw files and main text). These settings were kept same for all the wells. Therefore, transfection efficiency was computed as GFP+ Cell Count*100/Hoechst-Stained Count. These values were then normalized to the highest average transfection efficiency obtained (Cyc1 = 7.5%, N/P = 5) to obtain Figure 4a. It provides the information of *how many cells were transfected* overall.

b. Normalized GFP Cell Count: The 'Total Count' output from GFP target analysis was extracted from the raw data file and normalized to the highest GFP cell count obtained (Cyc1 = 7.5%, N/P = 5) for plotting Figure 4b.

c. GFP Mean Intensity: The 'AVG Target 1 Mean Intensity' output from GFP analysis was extracted from the raw data file for plotting Figure 4c. Target 1 Mean Intensity in Celigo is the *per-cell average fluorescence intensity* of the GFP channel, reported in arbitrary units, and is directly analogous to flow cytometry MFI. It provides the information of *how much GFP is present within each GFP+ cell.*

d. Cell Death (%): The 'Total Count' output from Hoechst target analysis was extracted from the raw data file for measuring the cell death. Cell death (%) was calculated as [(Y-X)*100/Y] where Y is the total count in the 'control' wells and X is the total count in treated wells.

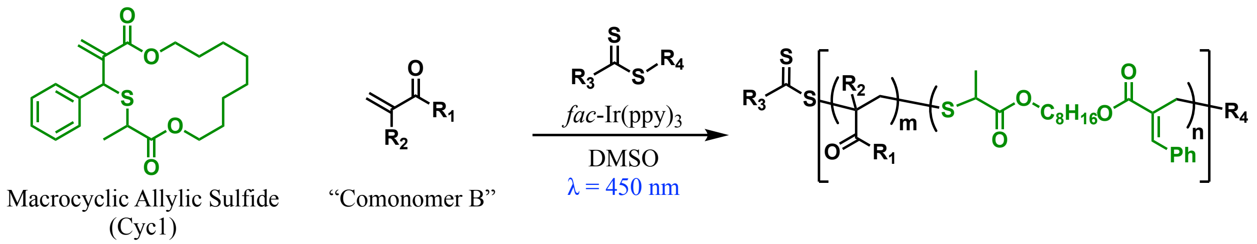

Figure S1. General synthetic scheme for copolymerization of macrocyclic allylic sulfide monomer Cyc1 with comonomer (*i.e.*, (meth)acrylates or (meth)acrylamides) via PET-RAFT polymerization. Cyc1 is able to participate in the PET-RAFT process via radical ring-opening cascade copolymerization (rROCCP).

P-HEMA-*co*-AEMAm-*co*-Cyc1 with $f_{Cyc1}{}^{0} = 0.1$

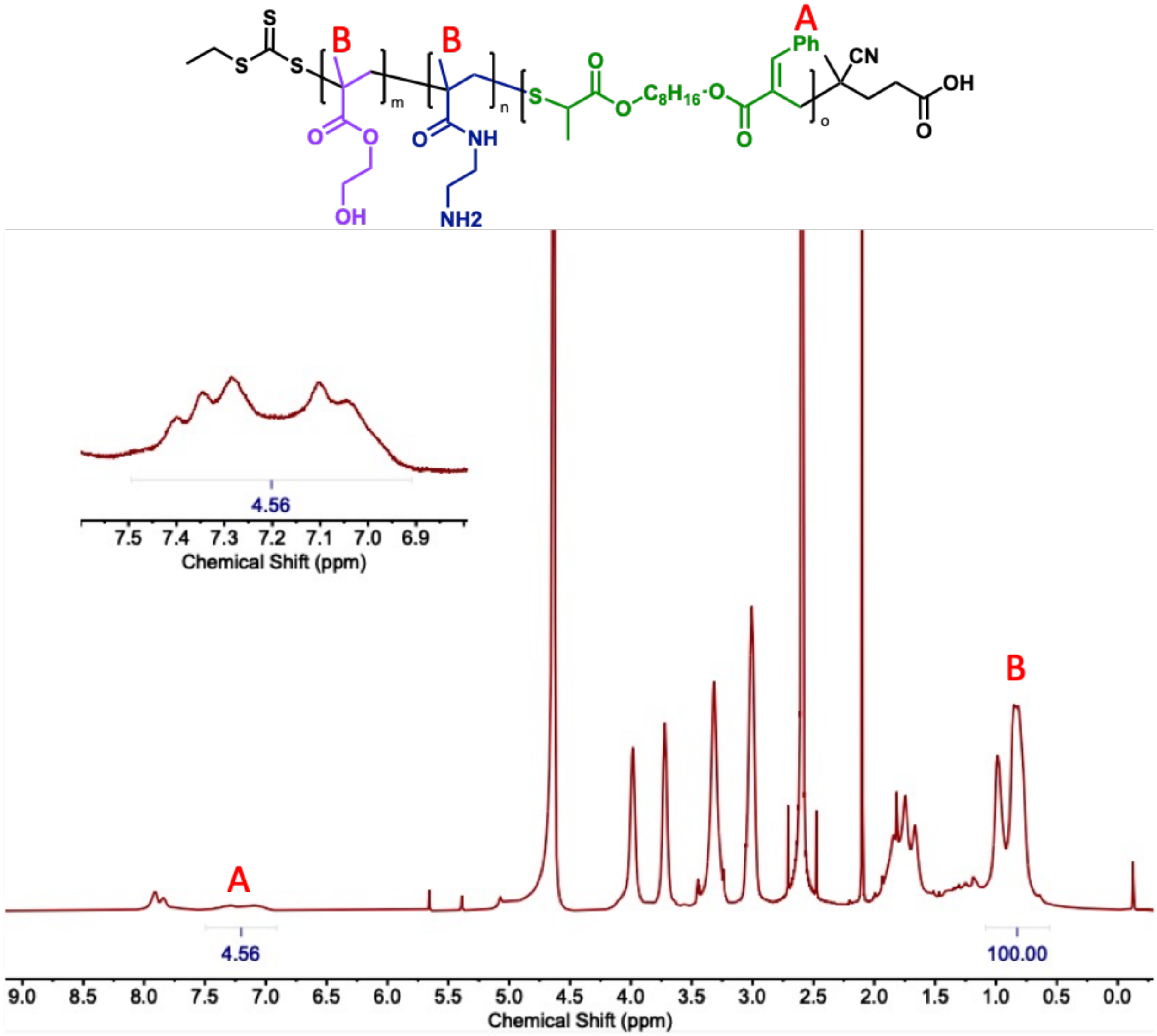

Figure S2. ^{1}H NMR in D_2O of degradable cationic copolymer prepared with Cyc1 feed ratio of 10 mol%. Degradable unit incorporation calculated by the integration of aromatic protons of the degradable comonomer Cyc1 (A) and methyl protons of HEMA and AEMAm comonomers (B): % incorp. = $[I_a/5]/([I_a/5] + [I_b/6])$ x 100%. As shown in the figure, % incorp. = (0.912/17.578) × 100% = 5.2%.

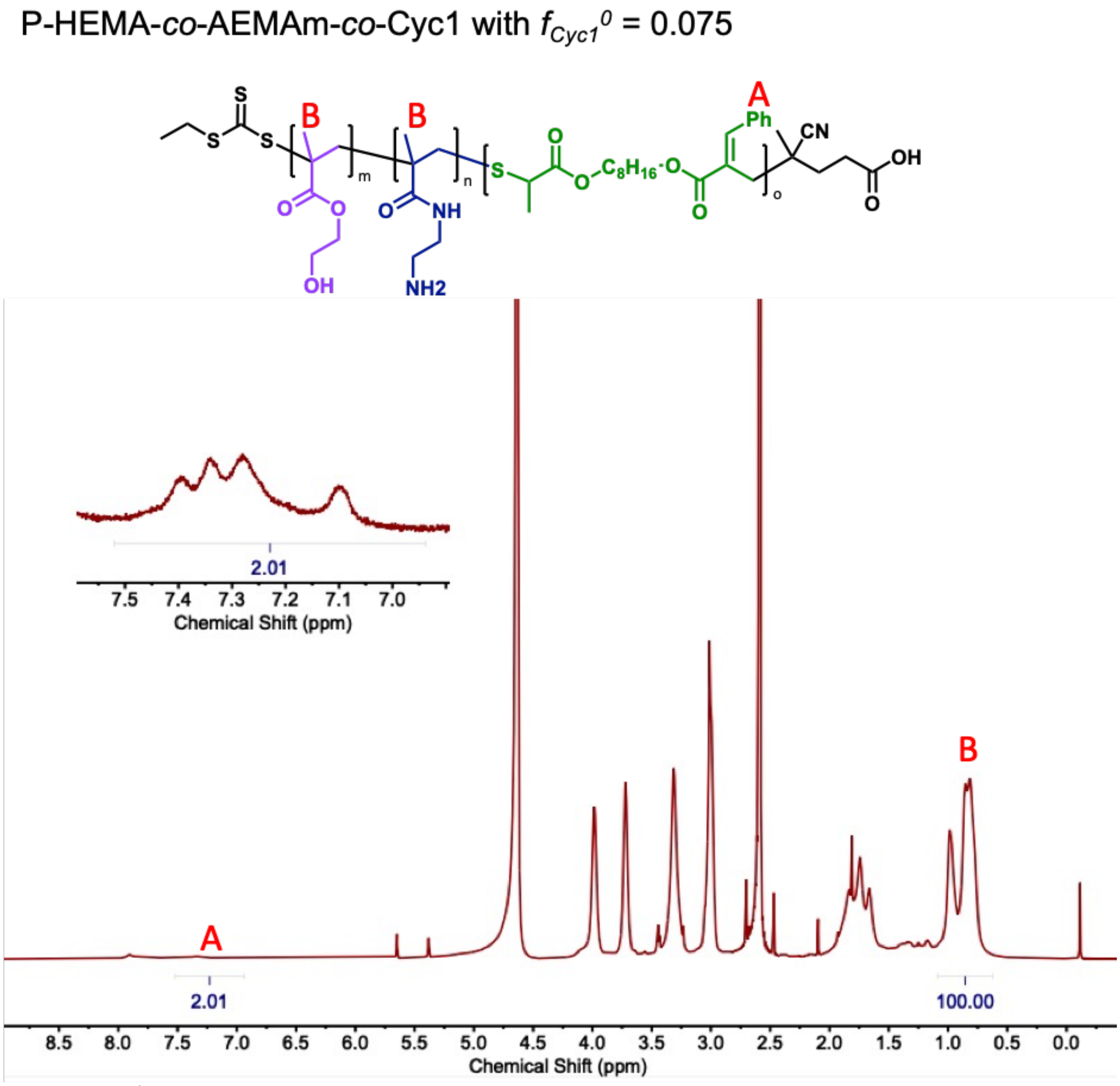

Figure S3. ^{1}H NMR in D_2O of degradable cationic copolymer prepared with Cyc1 feed ratio of 7.5 mol%. Degradable unit incorporation calculated by the integration of aromatic protons of the degradable comonomer Cyc1 (A) and methyl protons of HEMA and AEMAm comonomers (B): % incorp. = $[I_a/5]/([I_a/5] + [I_b/6])$ x 100%. As shown in the figure, % incorp. = (0.402/17.068) × 100% = 2.4%.

P-HEMA-*co*-AEMAm-*co*-Cyc1 with f_{Cyc1}^{0} = 0.05

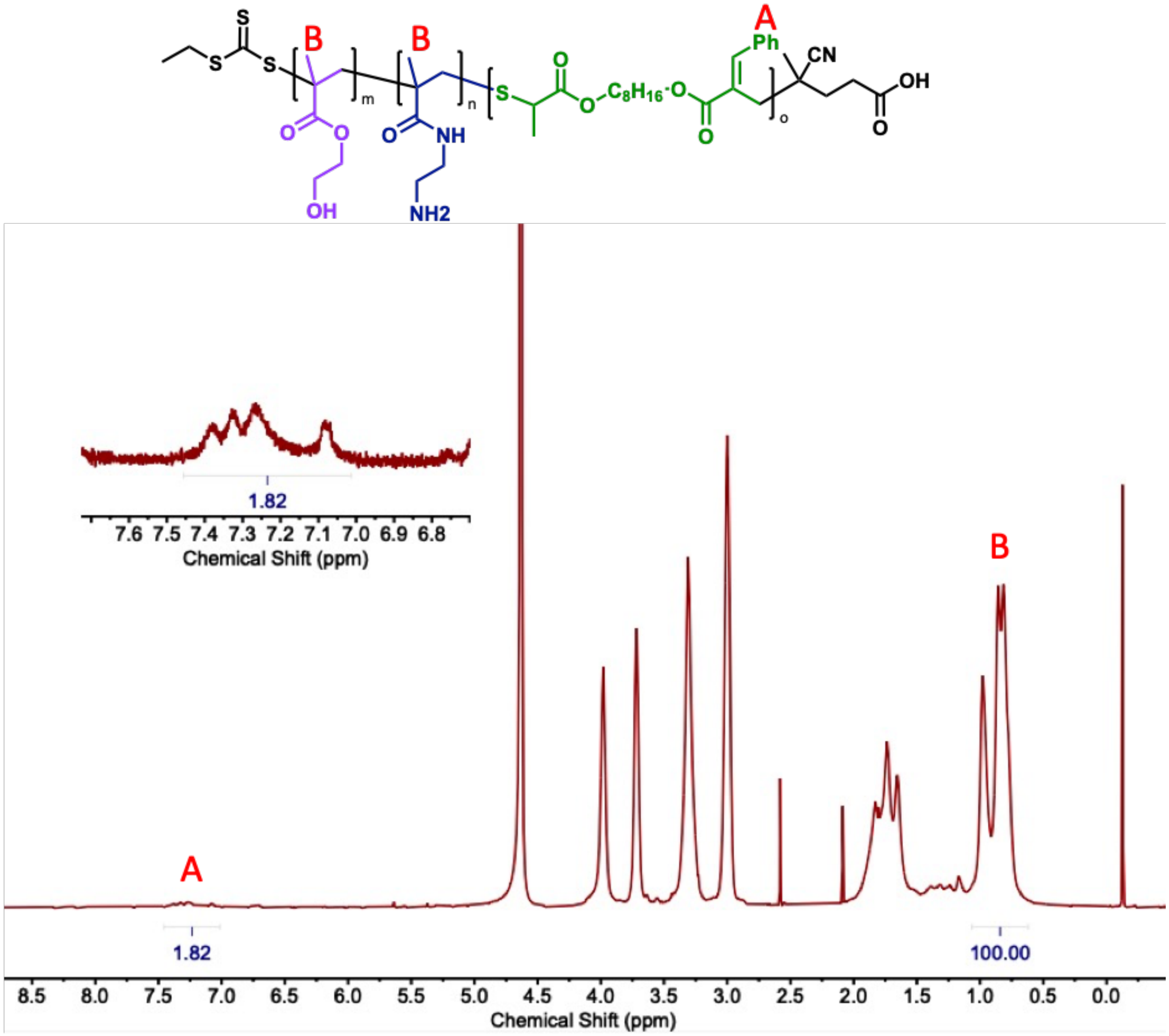

Figure S4. ^{1}H NMR in D_2O of degradable cationic copolymer prepared with Cyc1 feed ratio of 5 mol%. Degradable unit incorporation calculated by the integration of aromatic protons of the degradable comonomer Cyc1 (A) and methyl protons of HEMA and AEMAm comonomers (B): % incorp. = $[I_a/5]/([I_a/5] + [I_b/6])$ x 100%. As shown in the figure, % incorp. = (0.364/17.030) × 100% = 2.1%.

P-HEMA-*co*-AEMAm-*co*-Cyc1 with f_{Cyc1}^{0} = 0.025

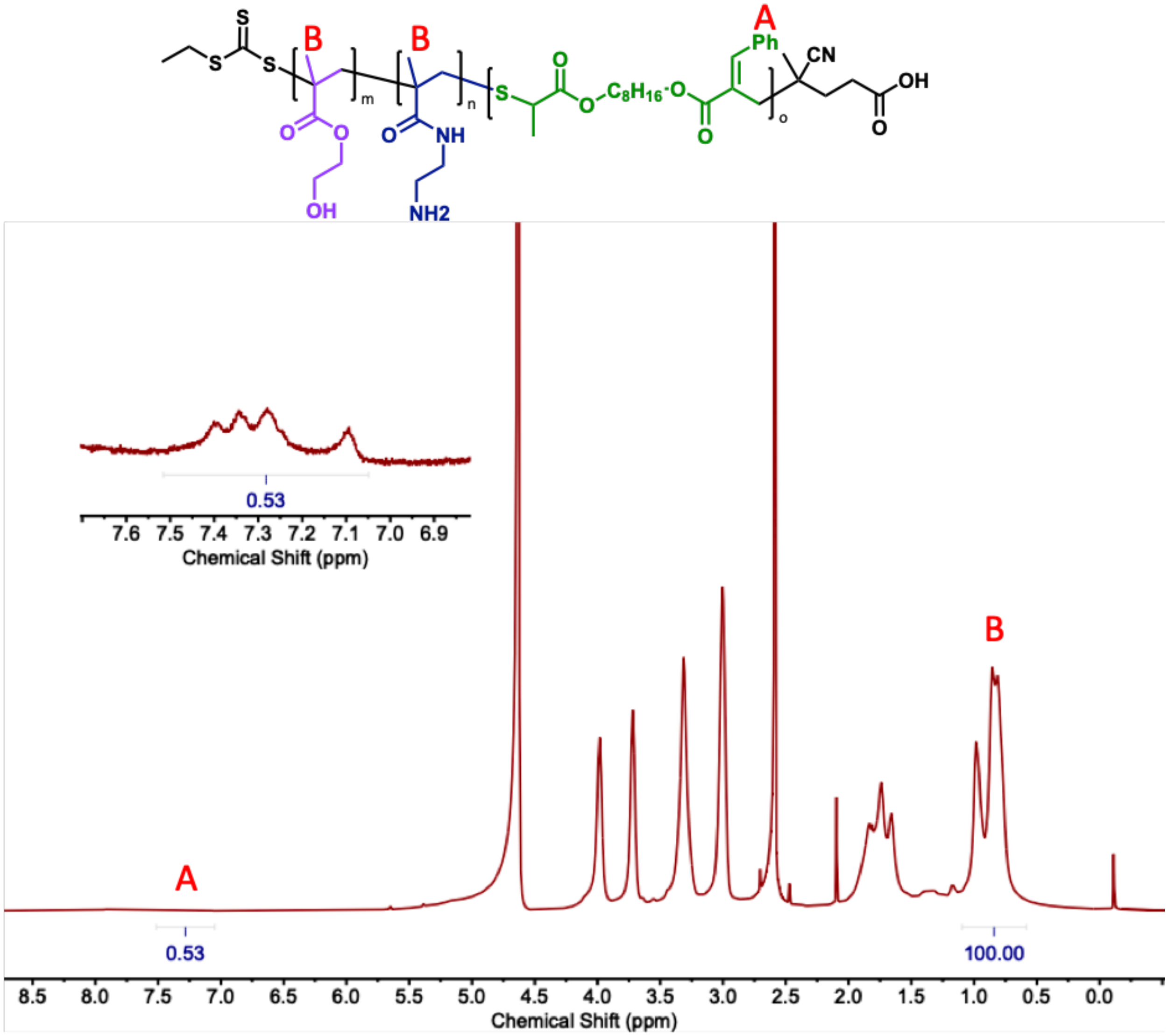

Figure S5. ^{1}H NMR in D_2O of degradable cationic copolymer prepared with Cyc1 feed ratio of 2.5 mol%. Degradable unit incorporation calculated by the integration of aromatic protons of the degradable comonomer Cyc1 (A) and methyl protons of HEMA and AEMAm comonomers (B): % incorp. = $[I_a/5]/([I_a/5] + [I_b/6])$ x 100%. As shown in the figure, % incorp. = (0.106/16.772) × 100% = 0.6%.

P-HEMA-*co*-AEMAm ($f_{Cyc1}{}^{0}$ = 0)

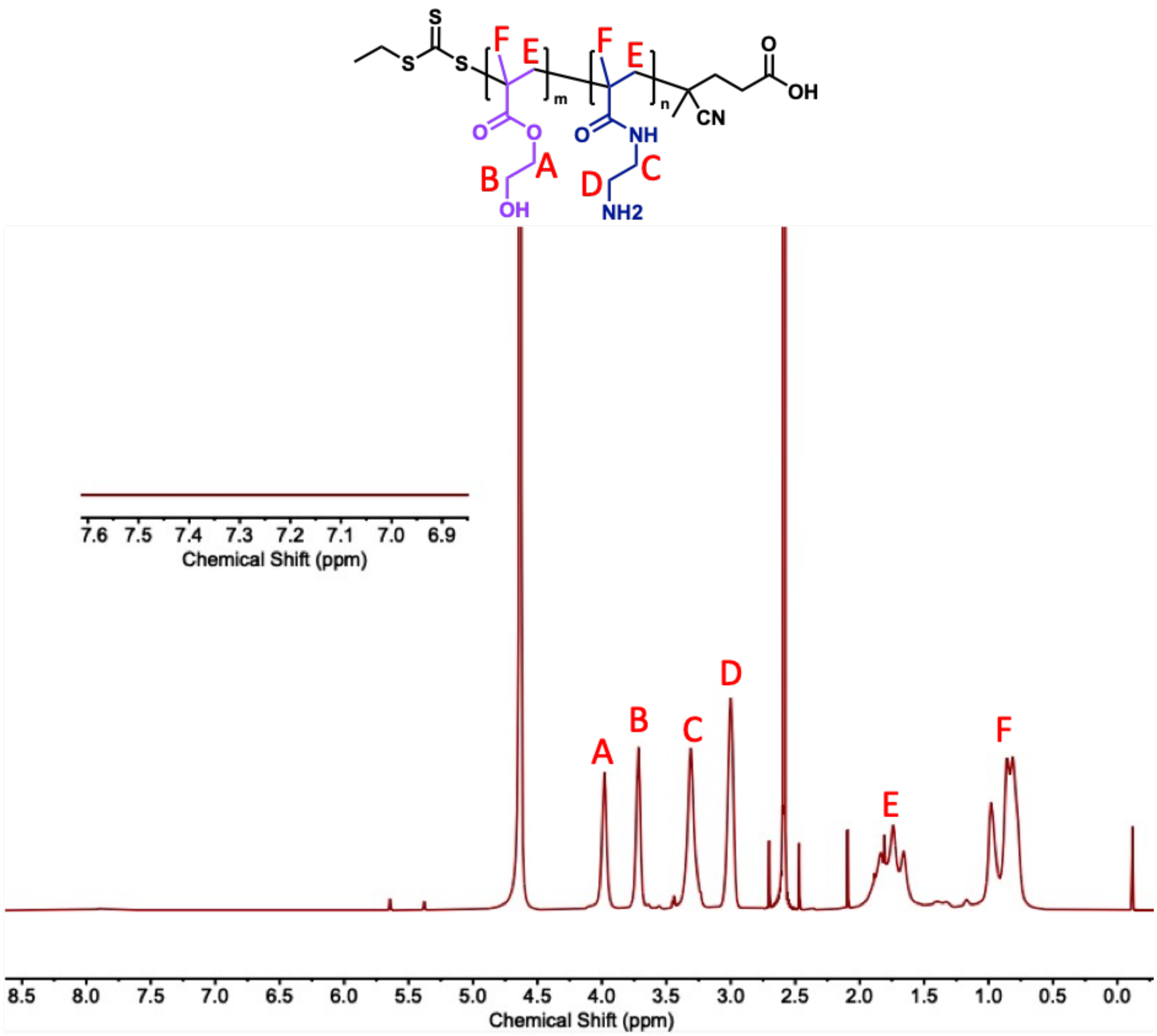

Figure S6. ^{1}H NMR in D_2O of non-degradable cationic copolymer prepared without Cyc1 in feed ratio.

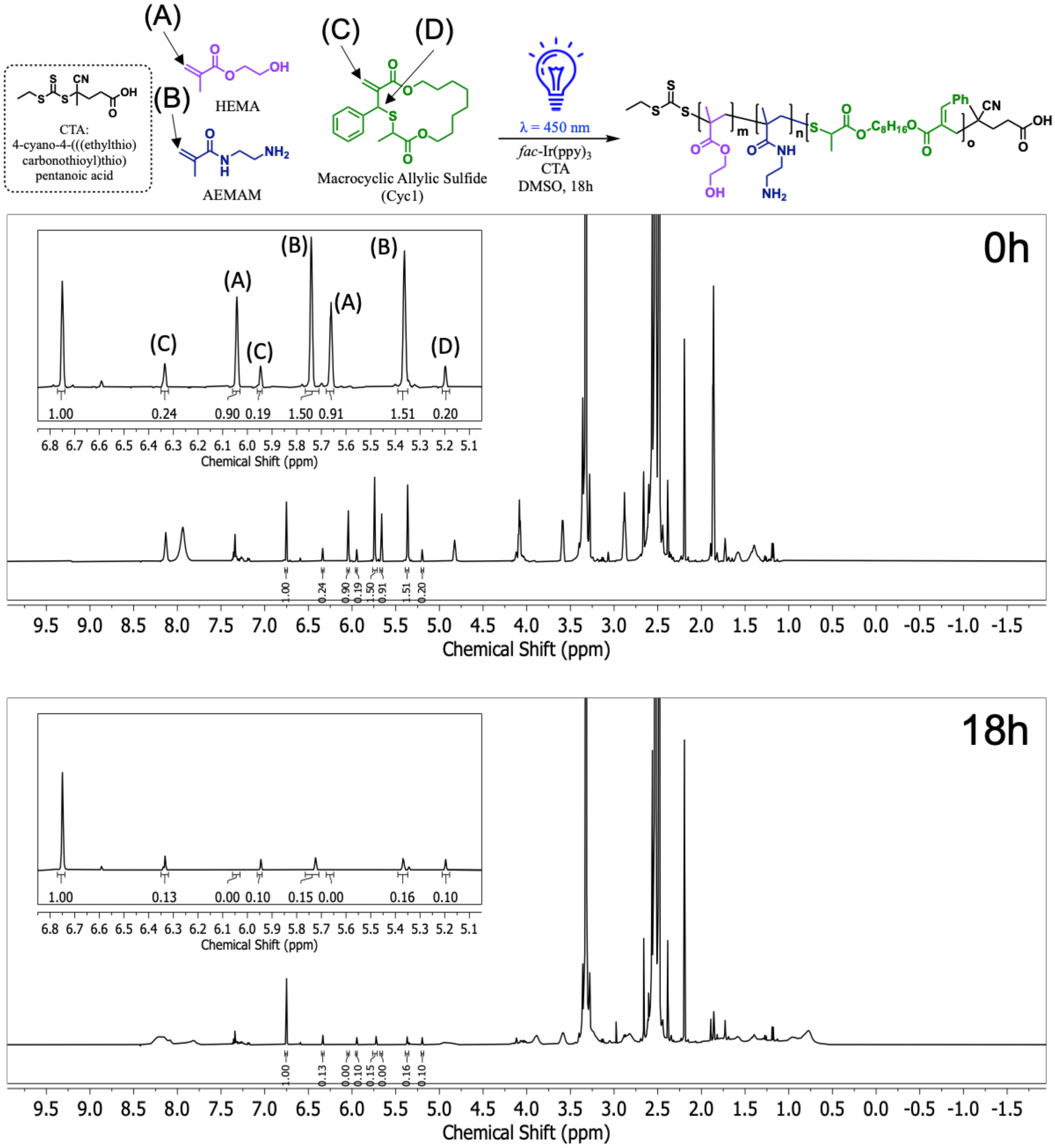

Figure S7. ^{1}H NMR (DMSO-d_6) of reaction mixture for degradable cationic copolymer **P1** ($f_{AEMAm}^0 = 0.6$, $f_{HEMA}^0 = 0.3$, $f_{Cyc1}^0 = 0.1$) at the start (0h, top) and end (18h, bottom) of the reaction. Integrals are assigned to vinyl protons of unreacted HEMA (δ = 6.04 ppm and δ = 5.66 ppm, "A"), vinyl protons of unreacted AEMAm (δ = 5.74 ppm and δ = 5.36 ppm, "B"), vinyl protons of unreacted Cyc1 (δ = 6.34 ppm and δ = 5.95 ppm, "C"), and allylic proton of unreacted Cyc1 (δ = 5.20, "D").

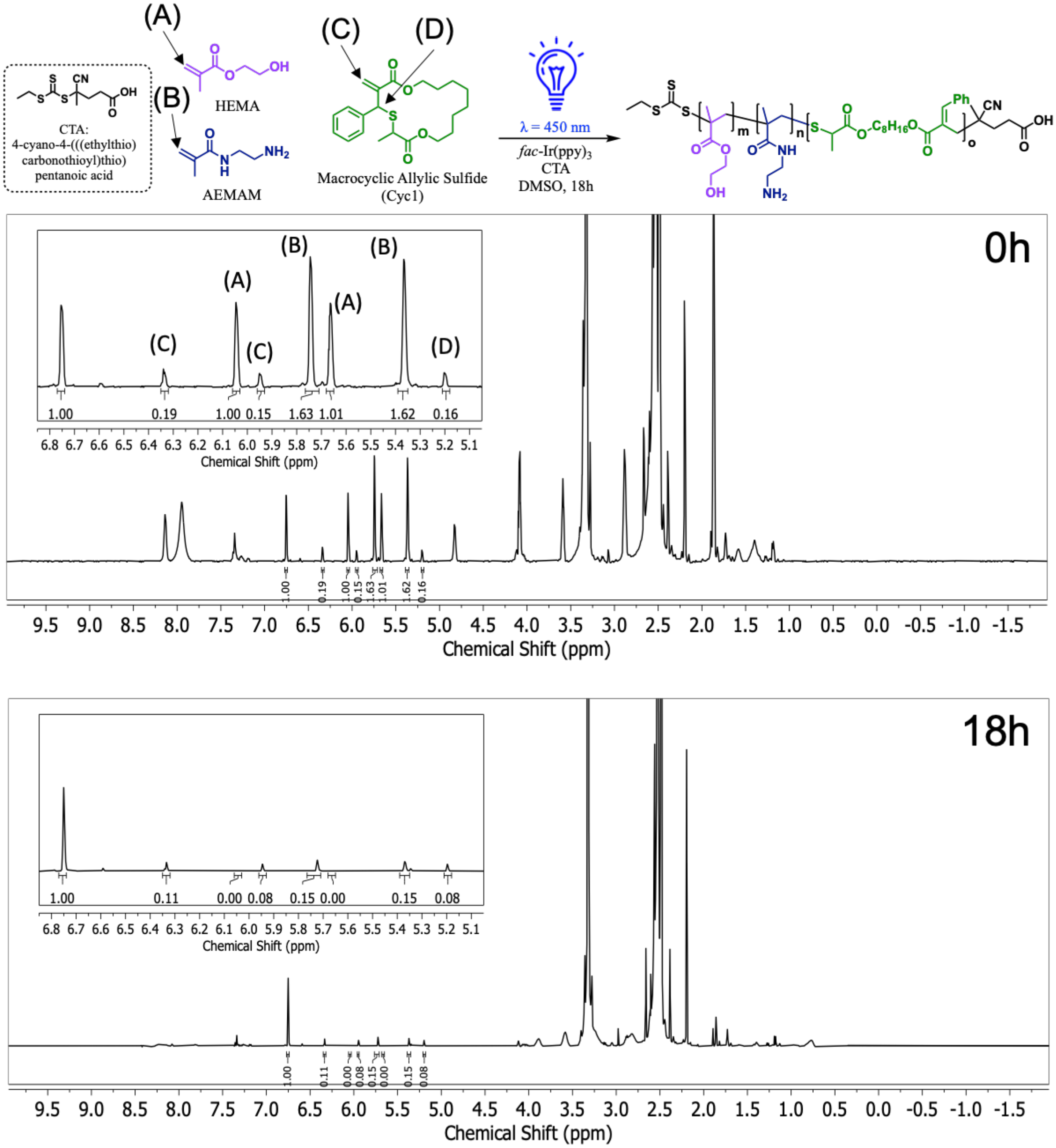

Figure S8. ^{1}H NMR (DMSO-d$_6$) of reaction mixture for degradable cationic copolymer **P2** ($f_{AEMAm}^0 = 0.6$, $f_{HEMA}^0 = 0.325$, $f_{Cyc1}^0 = 0.075$) at the start (0h, top) and end (18h, bottom) of the reaction. Integrals are assigned to vinyl protons of unreacted HEMA (δ = 6.04 ppm and δ = 5.66 ppm, "A"), vinyl protons of unreacted AEMAm (δ = 5.74 ppm and δ = 5.36 ppm, "B"), vinyl protons of unreacted Cyc1 (δ = 6.34 ppm and δ = 5.95 ppm, "C"), and allylic proton of unreacted Cyc1 (δ = 5.20, "D").

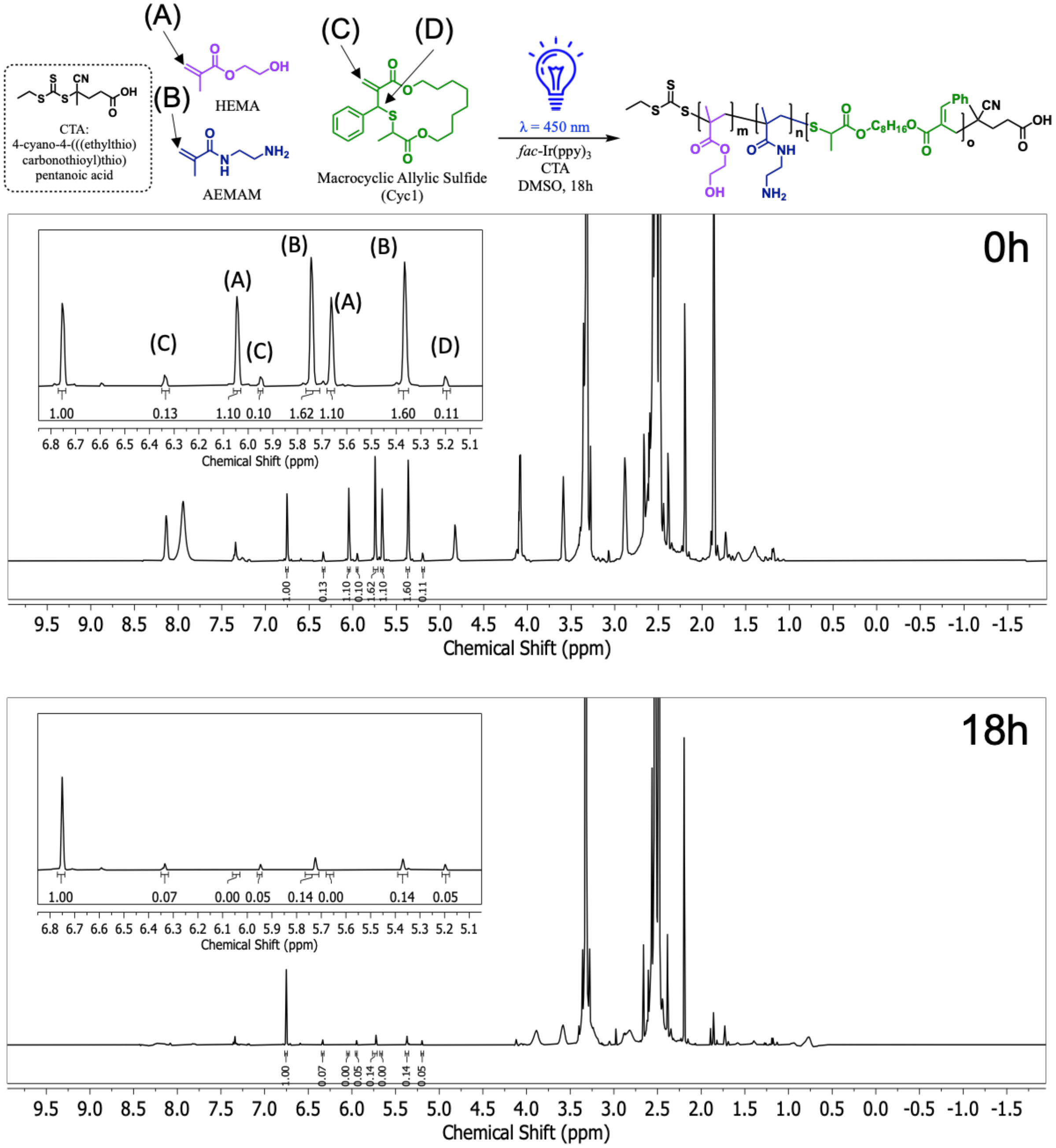

Figure S9. ^{1}H NMR (DMSO-d$_6$) of reaction mixture for degradable cationic copolymer **P3** ($f_{AEMAm}{}^0$ = 0.6, $f_{HEMA}{}^0$ = *0.35,* $f_{Cyc1}{}^0$ = *0.05)* at the start (0h, top) and end (18h, bottom) of the reaction. Integrals are assigned to vinyl protons of unreacted HEMA (δ = 6.04 ppm and δ = 5.66 ppm, "A"), vinyl protons of unreacted AEMAm (δ = 5.74 ppm and δ = 5.36 ppm, "B"), vinyl protons of unreacted Cyc1 (δ = 6.34 ppm and δ = 5.95 ppm, "C"), and allylic proton of unreacted Cyc1 (δ = 5.20, "D").

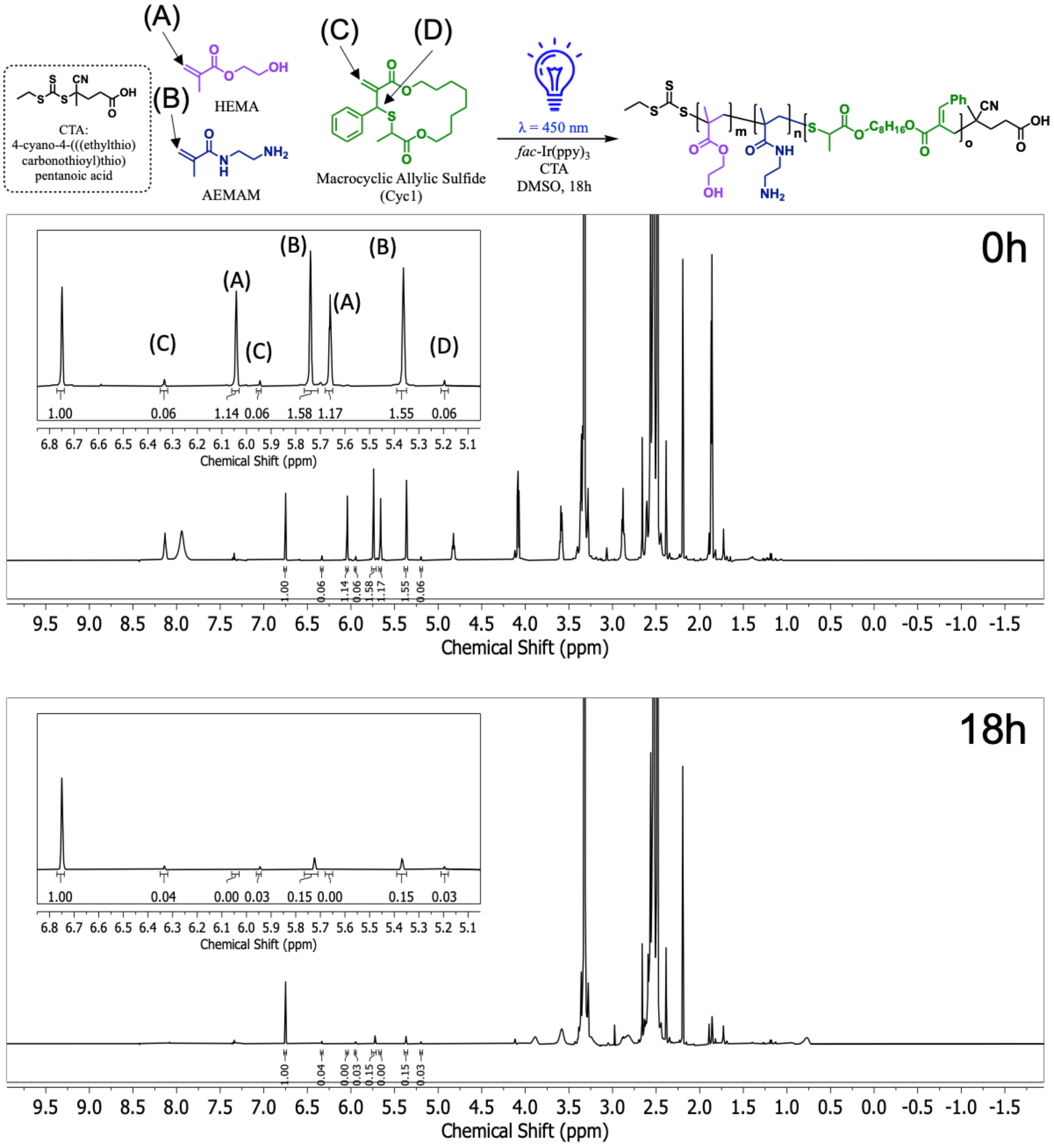

Figure S10. ^{1}H NMR (DMSO-d$_6$) of reaction mixture for degradable cationic copolymer **P4** ($f_{AEMAm}{}^0$ = 0.6, $f_{HEMA}{}^0$ = *0.375*, $f_{Cyc1}{}^0$ = *0.025)* at the start (0h, top) and end (18h, bottom) of the reaction. Integrals are assigned to vinyl protons of unreacted HEMA (δ = 6.04 ppm and δ = 5.66 ppm, "A"), vinyl protons of unreacted AEMAm (δ = 5.74 ppm and δ = 5.36 ppm, "B"), vinyl protons of unreacted Cyc1 (δ = 6.34 ppm and δ = 5.95 ppm, "C"), and allylic proton of unreacted Cyc1 (δ = 5.20, "D").

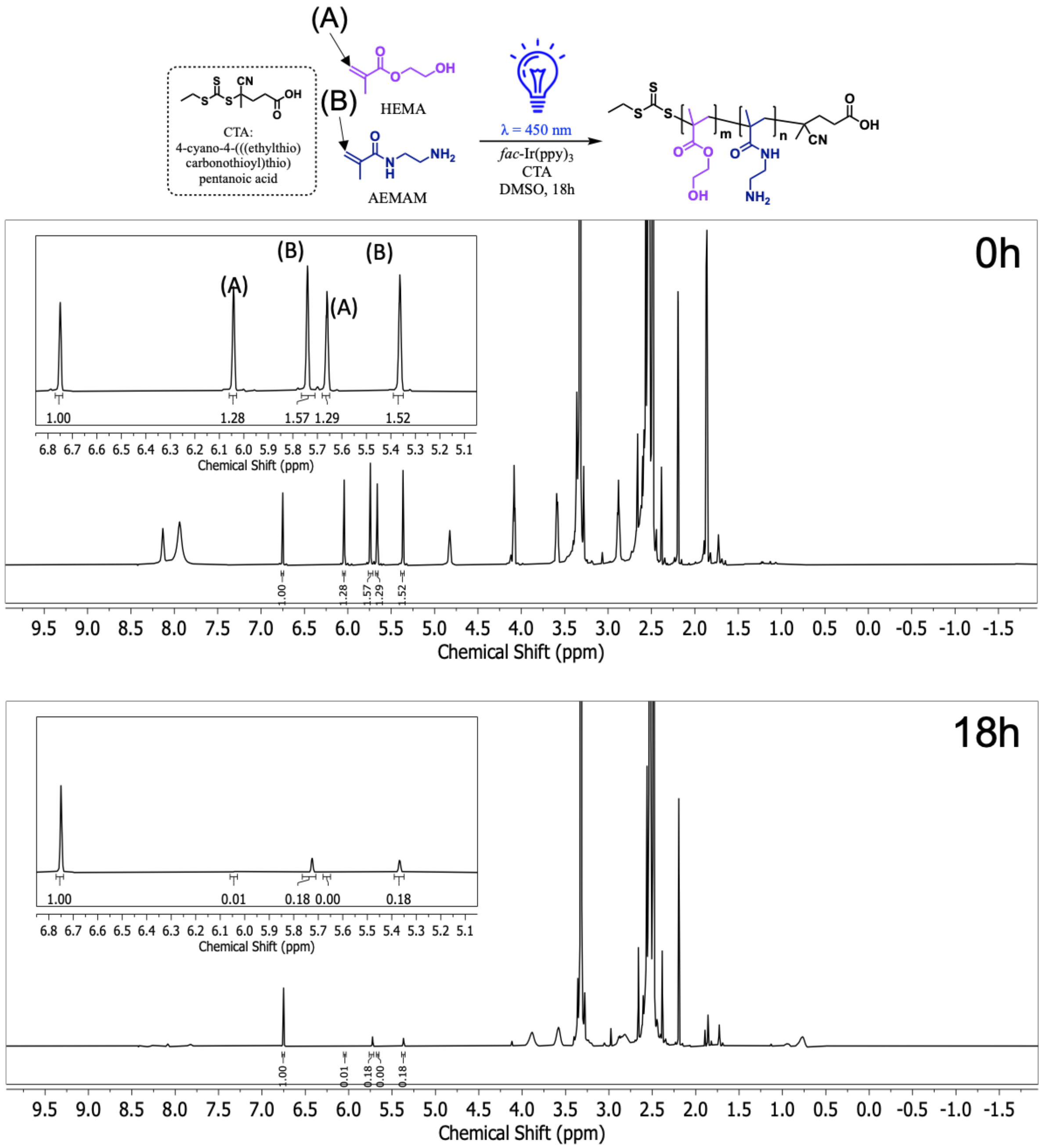

Figure S11. ^{1}H NMR (DMSO-d$_6$) of reaction mixture for non-degradable cationic copolymer **P5** ($f_{AEMAm}{}^0 = 0.6$, $f_{HEMA}{}^0 = 0.4$, $f_{Cyc1}{}^0 = 0$) at the start (0h, top) and end (18h, bottom) of the reaction. Integrals are assigned to vinyl protons of unreacted HEMA (δ = 6.04 ppm and δ = 5.66 ppm, “A”) and vinyl protons of unreacted AEMAm (δ = 5.74 ppm and δ = 5.36 ppm, “B”).

Table S1. Summary of monomer conversion of PET-RAFT reactions used to prepare polymer library (**P1-P5**) for *in vitro* transfection and cytotoxicity studies.

Poly ID	[M]:[CTA]	*Feed Ratio (mol%)*			*% Conversion*		
		HEMA	AEMAm	Cyc1	HEMA	AEMAM	Cyc1
P1	100	30	60	10	99.7%	89.8%	48.0%
P2	100	32.5	60	7.5	99.8%	90.9%	47.0%
P3	100	35	60	5	99.7%	91.1%	48.1%
P4	100	37.5	60	2.5	99.7%	90.5%	47.3%
P5	100	40	60	-	99.6%	88.3%	-

Monomer conversion was determined based on assigned integrals for vinylic or allylic protons for each of the three unique monomers. Integrals were normalized to mesitylene internal standard (δ = 6.75 ppm), and change in the normalized integral (100% - I_{final}/I_{inital}) was used to calculate monomer conversion. Percent conversion reported for a monomer represents the average change across all integrals assigned to protons for that monomer.

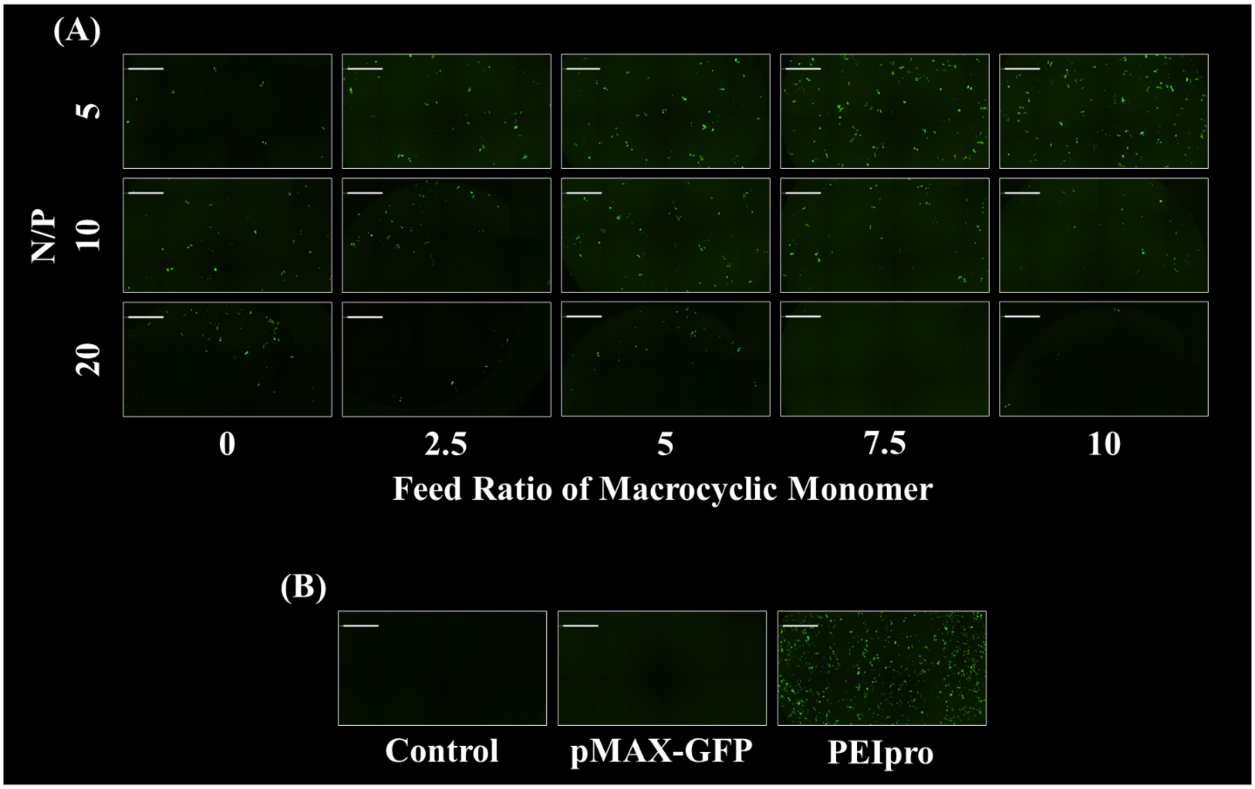

Figure S12 (A) Fluorescence microscopy of U-2 OS cells transfected with polyplexes formed with degradable and non-degradable polyplexes with varied feed ratio of the degradable residue and varied N/P ratios. (B) Images of U-2 OS control cells, cells transfected only with pMAX_GFP without any polymer delivery vehicle, and cells transfected with PEIpro. GFP expression was measured using target expression analysis with a Celigo Image Cytometer (Nexcelom Bioscience). Green fluorescence channel (483/536) was used to image the GFP expression with an exposure time of 10 ms. Celigo software was used for the automated image analysis that counts the GFP-positive cells and the mean intensity of GFP expression in each cell. Scale bar = 1 mm. Untreated cells and those treated with only the pMAX-GFP vector (i.e., no polymer) did not demonstrate any transfection.

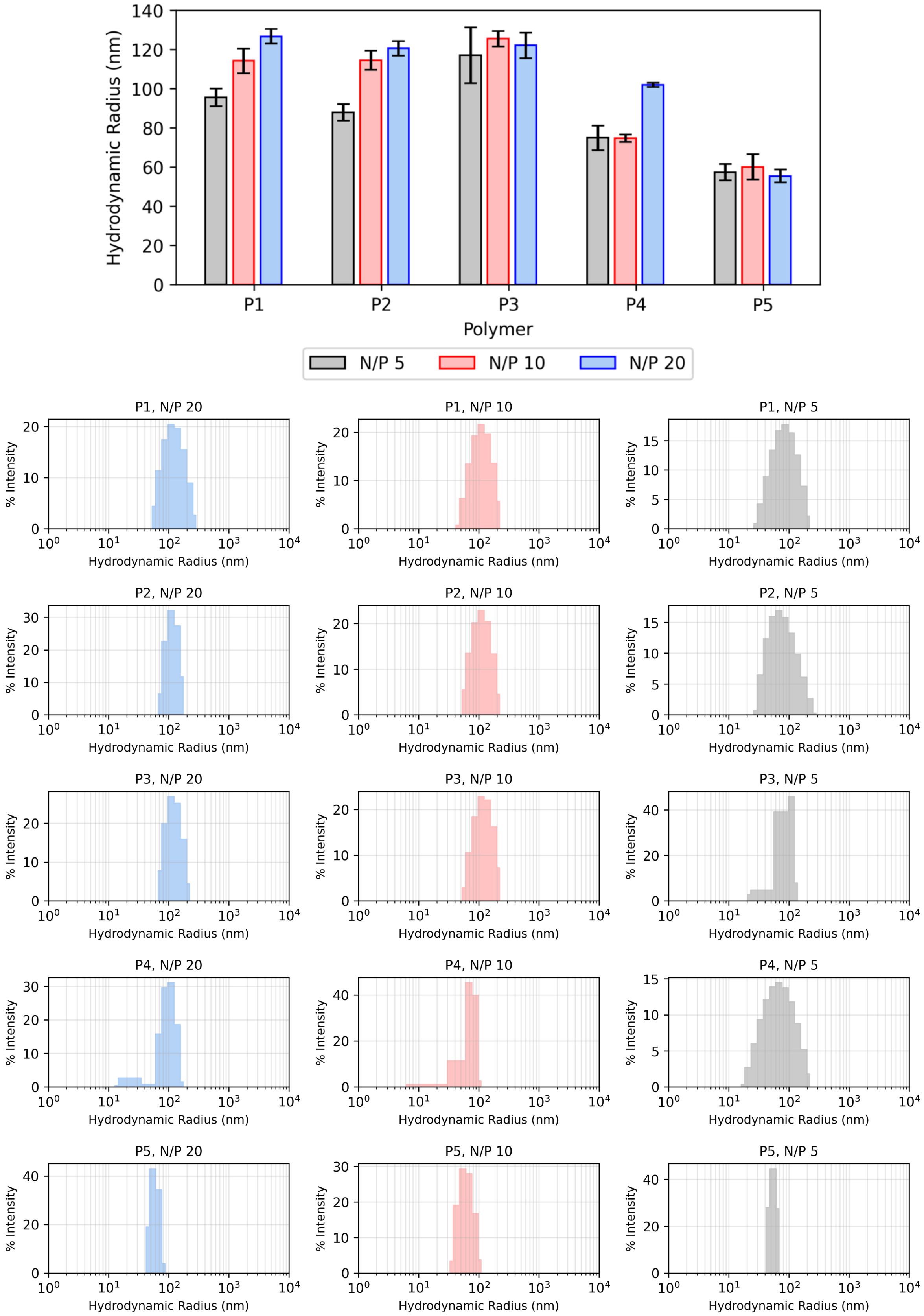

Hydrodynamic Radius (nm)
Polymer
P1
P2
P3
P4
P5
N/P 5
N/P 10
N/P 20
P1, N/P 20
P1, N/P 10
P1, N/P 5
P2, N/P 20
P2, N/P 10
P2, N/P 5
P3, N/P 20
P3, N/P 10
P3, N/P 5
P4, N/P 20
P4, N/P 10
P4, N/P 5
P5, N/P 20
P5, N/P 10
P5, N/P 5
% Intensity
Hydrodynamic Radius (nm)

Figure S13: Dynamic light scattering (DLS)-based characterization of polyplexes formed from polymer library. (Top) Summary of hydrodynamic radii for polyplex library. Polyplexes were formed in PBS and measured in triplicate. Data are reported as mean ± SD. (Bottom) Representative histograms of each polyplex demonstrating formation of well-defined particles with relatively narrow dispersities and a lack of large aggregates.

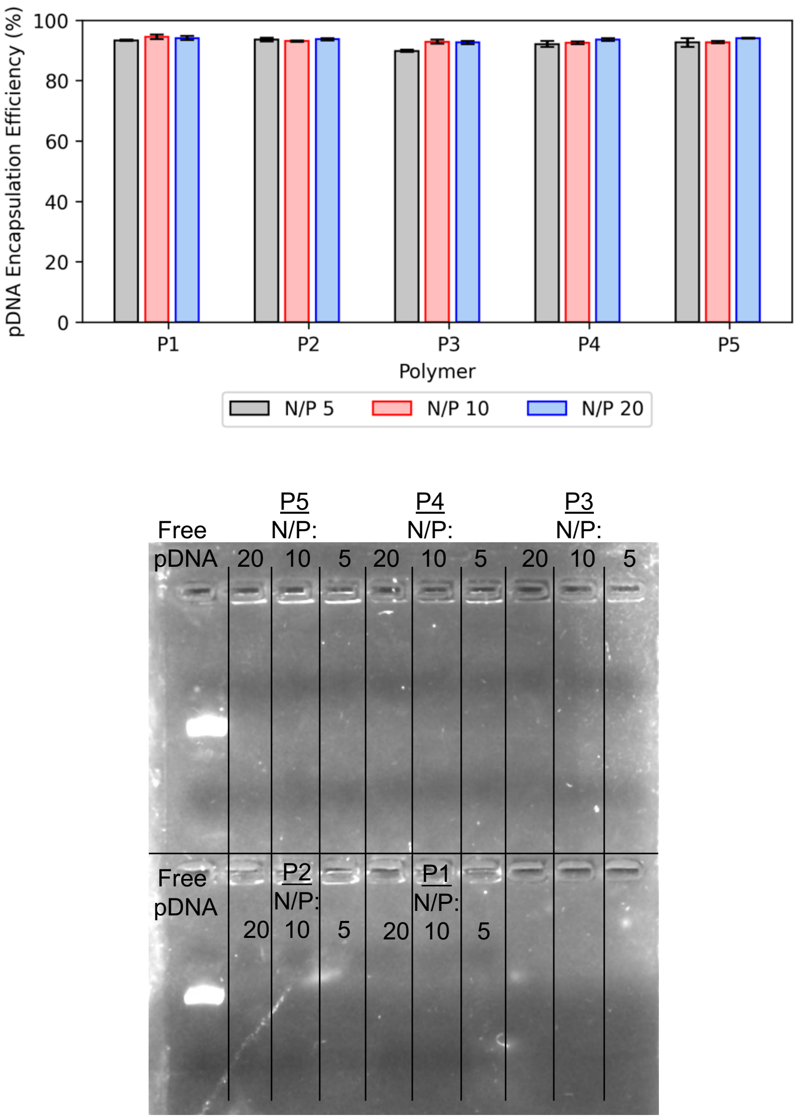

Figure S14: Complexation efficiency of cationic polymer library. (Top) Quantification of pDNA encapsulation efficiency as reported by a picogreen-based quantification assay. All polyplexes demonstrated high efficiency (>90%). (Bottom) Gel electrophoresis-based quantification of free pDNA after polyplex formation. No free pDNA is observed in the gel indicating near-quantitative incorporation of the pDNA at all N/P ratios tested.

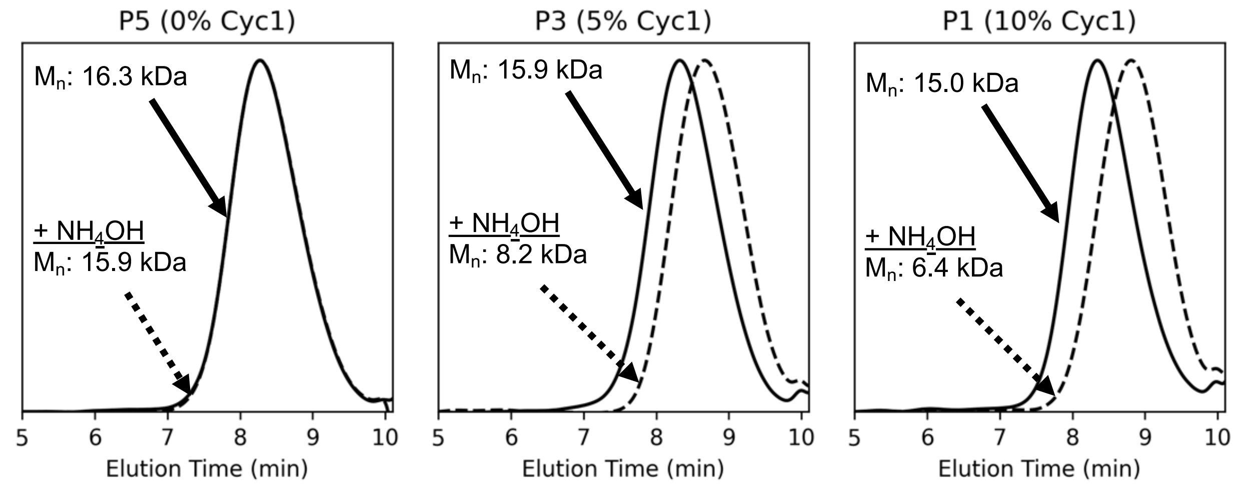

Figure S15: Backbone degradability of cationic copolymers. Polymers synthesized without the macrocyclic comonomer (P5) show no change in molecular weight after incubation with NH_4OH. In contrast, ester-containing copolymers (P1 and P3) saw significant shifts in response to chemical challenge. Furthermore, the degree of fragmentation increased for the construct with a greater number of ester residues (P1 vs P3), indicating degradability of the cationic polymer system could be tuned by altering the feed ratio of the cyclic comonomer Cyc1.
