# Supplementary figures and images for: Gene Delivery Mediated by Backbone-Degradable RAFT Copolymers
Source: Biomacromolecules. 2026 Feb 12;27(3):1846–56. doi: 10.1021/acs.biomac.5c01662 (PMC12977016; doi:10.1021/acs.biomac.5c01662)

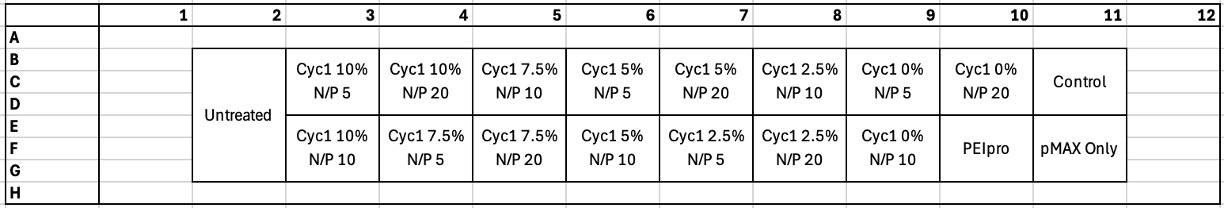

Supplement: Supplementary file 2 [file bm5c01662_si_002.zip › Celigo Raw Data/Plate Design.jpg]
